# Characterization of Cognitive Deficits in Rats Overexpressing Human Alpha-Synuclein in the Ventral Tegmental Area and Medial Septum Using Recombinant Adeno-Associated Viral Vectors

**DOI:** 10.1371/journal.pone.0064844

**Published:** 2013-05-21

**Authors:** Hélène Hall, Michael Jewett, Natalie Landeck, Nathalie Nilsson, Ulrika Schagerlöf, Giampiero Leanza, Deniz Kirik

**Affiliations:** 1 Brain Repair and Imaging in Neural Systems, Department of Experimental Medical Science, Lund University, Lund, Sweden; 2 Department of Life Sciences, Basic Research and Integrative Neuroscience, Centre for Neuroscience, University of Trieste, Trieste, Italy; Hertie Institute for Clinical Brain Research and German Center for Neurodegenerative Diseases, Germany

## Abstract

Intraneuronal inclusions containing alpha-synuclein (a-syn) constitute one of the pathological hallmarks of Parkinson's disease (PD) and are accompanied by severe neurodegeneration of A9 dopaminergic neurons located in the substantia nigra. Although to a lesser extent, A10 dopaminergic neurons are also affected. Neurodegeneration of other neuronal populations, such as the cholinergic, serotonergic and noradrenergic cell groups, has also been documented in PD patients. Studies in human post-mortem PD brains and in rodent models suggest that deficits in cholinergic and dopaminergic systems may be associated with the cognitive impairment seen in this disease. Here, we investigated the consequences of targeted overexpression of a-syn in the mesocorticolimbic dopaminergic and septohippocampal cholinergic pathways. Rats were injected with recombinant adeno-associated viral vectors encoding for either human wild-type a-syn or green fluorescent protein (GFP) in the ventral tegmental area and the medial septum/vertical limb of the diagonal band of Broca, two regions rich in dopaminergic and cholinergic neurons, respectively. Histopathological analysis showed widespread insoluble a-syn positive inclusions in all major projections areas of the targeted nuclei, including the hippocampus, neocortex, nucleus accumbens and anteromedial striatum. In addition, the rats overexpressing human a-syn displayed an abnormal locomotor response to apomorphine injection and exhibited spatial learning and memory deficits in the Morris water maze task, in the absence of obvious spontaneous locomotor impairment. As losses in dopaminergic and cholinergic immunoreactivity in both the GFP and a-syn expressing animals were mild-to-moderate and did not differ from each other, the behavioral impairments seen in the a-syn overexpressing animals appear to be determined by the long term persisting neuropathology in the surviving neurons rather than by neurodegeneration.

## Introduction

Clinically, Parkinson's disease (PD) is characterized by the presence of motor symptoms, resulting from the loss of striatal dopamine (DA) following neurodegeneration of dopaminergic cells in the substantia nigra (SN) [1], [2], [3]. However, the clinical expression of the disease is more heterogeneous as patients suffer from a variety of additional non-motor symptoms, including sleep disturbances, olfactory deficits, cognitive impairment, neuropsychiatric disorders, and autonomic dysfunction [4], [5]. In particular, the cognitive deficit seen in PD causes disturbances in both executive functions and memory [6] and may lead to dementia in 30 to 40% of the patients as the disease progresses [7], [8].

The neurodegeneration observed in PD is not limited to dopaminergic neurons of the SN. In fact, in some cases, other dopaminergic cells located in specific nuclei of the A10 group also degenerate [9]. Likewise, non-dopaminergic cell groups appear to be affected, including those in the cholinergic, serotonergic and noradrenergic systems [see [10] for an extensive review]. For example, a loss of cholinergic neurons in the basal forebrain and a profound decrease in cortical and hippocampal choline acetyltransferase activity have been reported in PD [11], [12], [13]. Importantly, the alteration of the cholinergic system is more severe in cognitively impaired PD patients [13] as well as in demented PD patients [14].

In light of such results, it is likely that both the dopaminergic and cholinergic deficits seen in PD may be pivotal mechanisms in the development of a cognitive dysfunction. In fact, a previous study performed in rats showed that toxic lesion of dopaminergic neurons in the ventral tegmental area (VTA) was sufficient to induce reference memory impairment and that simultaneous toxin-induced loss of dopaminergic neurons in the VTA and of cholinergic neurons in the medial septum/vertical limb of the diagonal band of Broca (MS/vDBB) was necessary and sufficient to induce an additional deficit in working memory. This suggested that the integrity of the mesocorticolimbic dopaminergic pathway is critical for memory functions and that it might act synergistically with the septohippocampal cholinergic pathways to regulate certain aspects of learning and memory [15].

One of the main pathological features of PD is the presence of Lewy bodies (LB) - intracellular inclusions of aggregated proteins, in which alpha-synuclein (a-syn) accumulates [16]. LB are not restricted to the SN and are found throughout the brain. In fact, according to the hypothesis put forward by Braak and colleagues, the development of pathology in midbrain structures is preceded by its occurrence in the lower brainstem, and in the later stages of the disease spreads to cortical structures [17].

Overexpression of wild-type and mutant a-syn in the SN using viral vectors has been successfully used to model the dopaminergic cell loss and associated cellular and axonal pathology seen in PD, along with significant motor behavioral impairment in rats [18], [19], [20] and non-human primates [21], [22]. Interestingly, overexpression of mutant a-syn in the VTA in rats can also induce mild DA-dependent motor impairment [23]. In that study, VTA dopaminergic neurons were resistant to the mutant protein, suggesting that dysfunctional but surviving a-syn overexpressing neurons might underlie the development of behavioral impairment after transduction of dopaminergic neurons in the VTA region [23].

The aim of the present study was to examine the pathological effects of combined overexpression of human wild-type a-syn in the VTA and MS/vDBB in rats, two regions rich in dopaminergic and cholinergic neurons, respectively, and to further investigate whether this was associated with patterns of specific learning and memory deficits.

## Materials and Methods

### AAV vector design and production

The viral vectors used in this study were recombinant AAV serotype 5 (rAAV5) vectors carrying the coding sequence for human wild-type a-syn or for the green fluorescent protein (GFP). Plasmids were generated, expressing either a-syn or GFP, under the human synapsin 1 promoter. The cDNA for both genes was followed by a late SV40 poly-A sequence. The trafficking of the mRNA was improved by addition of a woodchuck hepatitis virus post-transcriptional regulatory element (WPRE). To terminate transcription an early SV40 poly-A sequence was used. The AAV vectors were produced with a double-transfection method with the appropriate transfer plasmid and the helper plasmid containing the essential adenoassociated viral packaging genes, as described previously [24]. They were thereafter purified by iodixanol step gradients and Sepharose Q column chromatography. The purified viral vector suspension was tittered with TaqMan quantitative PCR with primers and probes targeted toward the WPRE sequence. The final titer for vectors encoding a-syn and GFP genes was 3.2×10^13^ and 3.7×10^13^ genome copies/ml, respectively.

### Animals and experimental groups

32 adult female Sprague Dawley rats, weighing 225–250 g at the beginning of the experiment, were purchased from Charles River (Schweinfurt, Germany). All animals were housed 2–3 per cage and kept under a 12 h light/12 h dark cycle with *ad libitum* access to food and water (except during the staircase test where the animals were on a restricted food intake, as described below). All the experimental procedures were performed with approval from the Swedish Board of Agriculture (Jordbruksverket) and carried out in compliance with the rules of the Ethical Committee for Use of Laboratory Animals in the Lund-Malmö region. All efforts were made to minimize the number of rats used and their suffering.

Animals were allocated to one of the three groups to receive AAV vectors encoding human a-syn (n = 15), AAV vectors encoding GFP (vector control group; n = 7) or followed as normal controls (n = 10).

### Surgical procedures

All surgical procedures were performed under anesthesia using intraperitoneal injection of a 20∶1 mixture of fentanyl and medetomidine (Domitor; Apoteksbolaget, Sweden). The animals were placed in a stereotaxic frame (Stoelting, Wood Dale, IL, USA) and an incision was made on the skin overlying the skull. The anterioposterior (AP) and mediolateral (ML) coordinates of the injection sites were calculated from bregma and the dorsoventral (DV) coordinates from the dural surface, according to the stereotaxic atlas of Paxinos and Watson [25]. A 5 µl Hamilton syringe fitted with a glass pipette (OD: 60–80 µm) was used for the injections. To target the MS/VDBB, the animals received a total of 4 µl of either rAAV5-GFP (n = 7) (GFP group) or rAAV5- α-syn (n = 15) (a-syn group) vector, divided into two 1 µl-deposits on each side of the midline, using the following coordinates (all in mm): AP = +0.6; ML = ±1.5; DV = −8/−7, at an angle of 13° in the coronal plane and with the tooth bar set to −3.3. Once the first deposit was injected in the MS/VDBB, the needle was left in position for 2 min before being slowly moved 1 mm upward for the second deposit. After injection of the second deposit, the needle was left in place for 2 min, slowly moved 1 mm upward and kept in place for an additional 1 min before it was slowly retracted from the brain. In addition, an injection of 1.5 µl of either rAAV5-GFP (GFP group) or rAAV5-a-syn (a-syn group) vector was made in the midline VTA at the following coordinates: AP = −6.1; ML = −1.6; DV = −7.3 using the same angle and tooth bar position as for the MS/VDBB injections. After completion of the injection in the VTA, the needle was kept in place for 5 min before being slowly retracted from the brain. At both sites, injections were made at a rate of 0.4 µl/minute.

### Behavioral tests

**Figure 1:**
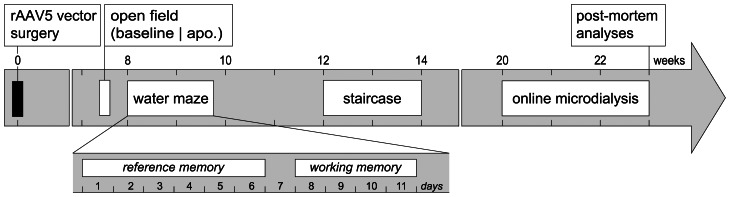
Time course of the experiment. Recombinant AAV serotype 5 (rAAV5) vectors encoding either human a-syn (n = 15) or GFP (n = 7) were injected in the ventral tegmental area (VTA) and medial septum/vertical limb of the diagonal band of Broca (MS/vDBB). Naive animals (n = 10) served as normal controls. All animals were submitted to behavioral assessment. Open field activity was tested at baseline condition and after subcutaneous injection of 0.1 mg/kg of apomorphine (apo). After a two-day washout period, animals were tested in the Morris water maze to evaluate learning and memory. To assess reference memory, the animals were given four trials per day over six consecutive days. Starting 2 days after the completion of reference memory test, the working memory of the animals was tested using a radial arm water maze. The animals were given five trials per day over four consecutive days. To assess motor learning, the animals were trained in the staircase test for eleven consecutive days. Twenty-three weeks post-AAV injection, animals were submitted to online microdialysis coupled to HPLC detection (OMD) to quantify in real time neurotransmitters levels in the hippocampus. Upon completion of the OMD session, animals were perfused and brains processed for histological analysis.

For assessment of cognitive and sensory-motor functions, the animals underwent a battery of behavioral tests administered sequentially (Fig. 1).

#### Open field activity (weeks 7–8)

The animals were subjected to open-field activity test in order to assess horizontal locomotor activity, using plexiglas boxes (40×40×38 cm) equipped with a 16×16 infrared photobeam system controlled by the Flexfield Software (San Diego Instruments, San Diego, CA, USA). On the testing day, baseline activity was first recorded for 1 h. Then, the animals received a subcutaneous (sc) injection of low dose apomorphine (0.1 mg/kg in 0.02% ascorbic acid) and their activity was monitored for an additional 1 h period. The average numbers of beam breaks per min counted between 30 and 60 min at baseline or between 5 and 35 min after the apomorphine injection were used as the testing variables between groups.

#### Water maze test

A modified version of the water maze task originally developed by Morris [26] was used to assess spatial learning and memory. The maze consisted of a circular tank (180 cm in diameter), filled with room temperature water, made opaque by the addition of non-toxic white paint. Extra-maze distal cues were positioned on the walls around the tank. A 15 cm-wide circular platform was fixed to the bottom of the tank and submerged 2 cm below the water surface to remain invisible to the animals. Two different paradigms, designed to evaluate reference and working memory, were implemented.

In the version of the test assessing reference memory, the animals were given four trials per day over six consecutive days. Four orientation points indicated as north (N), west (W), south (S) and east (E), served as starting positions, while the platform remained in the same position (SW quadrant). The sequence of release positions was changed every day. In each trial day, the rats were released from a different position and given 60 sec to locate the platform and climb onto it. Animals were given an inter-trial time of 20 sec, during which they remained on the platform. The latency to find the platform was recorded using a computer-based video tracking system (EthoVision 3.1.13; Noldus, Wageningen, The Netherlands). A probe trial was performed at the end of the training session, in which the platform was removed from the pool. Rats were allowed to swim for 60 s and the time spent in each quadrant as well as the number of annulus (defined as a circular zone surrounding where the platform was) crossings were recorded.

Two days after completing the reference memory task, the animals were submitted to the working memory version of the test, in which a radial arm water maze featuring six radially-distributed swimming arms (numbered 1 to 6) was used. The platform was placed at the end of an arm and its position was changed every day (but kept constant throughout a given day). The animals were given five trials per day over four consecutive days. In each trial, the rats were released from a different position and given 60 sec to locate the platform and climb onto it. The latency to locate the platform was recorded. In addition, the difference in average latency on days 3–4 between trials 1 and 2, calculated as percentage of trial 1, was used as an additional estimate of the animal's performance (savings).

#### Staircase test

In order to assess striatum-dependent motor learning, the paw-reaching task (or staircase test) was administered to all animals, as previously described [27]. Rats were deprived of their food two days before initiating the test and kept on a restricted food intake (6–8 g/day) throughout the testing phase (with food administered after the daily test session). The animals were trained for 20 min/day in Plexiglas boxes (285×60×90 mm) holding a double staircase divided by a central platform (35 mm). Four steps on each side (steps 2–5) were baited with ten sugar pellets (total 40 sugar pellets/side). Day 1 of analysis was defined as the first day the rats started retrieving pellets, typically on the 2^nd^ or 3^rd^ day of testing. At the end of the session, the number of pellets left on each step as well as the number of pellets displaced were counted to calculate the total number of pellets retrieved and the number of pellets missed (errors). The total number of pellets eaten was calculated as the difference between number of pellets retrieved and errors.

### Online Microdialysis coupled to HPLC detection

Rats were anesthetized with 2% isoflurane mixed with O_2_ and N_2_ and placed in a stereotaxic frame. The microdialysis probes (membrane length: 3 mm, membrane diameter: 0.5 mm, molecular weight cut-off 20 kDa; CMA Microdialysis, Solna, Sweden) were inserted into the ventral hippocampus at AP = −5,5 mm; ML = −4.8 mm relative to bregma and DV = −5.5 mm from the dural surface, with the tooth bar set to −3.3 mm. The probes were connected to a syringe infusion pump (Model 100; CMA Microdialysis, Solna, Sweden) and perfused at 0.85 µl/min with modified Ringer solution (147 mM NaCl, 3 mM KCl, 2.2 mM CaCl_2_, pH 6.4) containing neostigmine bromide (5 µM) for estimation of basal neurotransmitter levels or with enriched KCl solution (51 mM NaCl, 100 mM KCl, 2.2 mM CaCl_2_, pH 6.4) containing neostigmine bromide (5 µM) for measurement of neurotransmitter release. Preliminary analyses run on test animals showed that without neostigmine in the perfusion medium, ACh was not detectable in the hippocampus. Adding 1 µM of neostigmine in the hippocampus was also not sufficient to detect ACh. The outlet of the probes was connected to a 14-port injector valve and the dialysates split into two 8-µl loops resulting into two separate flow paths for HPLC analysis. Instrumentation and methods were developed and remodeled from a previous description to accommodate for parallel analysis of ACh and monoamines [28]. After one hour of equilibration, baseline samples were analyzed for one hour before the perfusate was changed to an enriched KCl Ringer solution for 12 minutes and then switched back to the normal Ringer solution. Dialysates were analyzed for an additional hour after KCl challenge. After collection of the last sample, the probe was withdrawn and the animals perfused as described below for histological analysis.

Monoamines and acetylcholine (ACh) were directly and simultaneously analyzed with electrochemical detection on an Alexys 100 LC-EC system (Antec Leyden, Zoeterwoude, The Netherlands) in 12-minute time bins. Both analytical columns and detector cells were kept at 35°C in a column oven. To analyze monoamines (NA, 5HT), the mobile phase (100 mM phosphoric acid, 50 mM citric acid, 0.1 mM EDTA, 8 mM NaCl, 350 mg/l OSA, 15% MetOH, pH 3.8) was run through a C18 reverse phase 1 mm×150 mm column with 3 µm particle size (ALF 115) at a flow rate of 70 µl/min. After separation on the column, the monoamines were detected electrochemically with a DECADE II detector coupled to a VT-03 flow cell (Antec Leyden, The Netherlands) with a glassy carbon working electrode (potential set to +0.75 V versus Ag/AgCl salt bridge reference electrode). ACh levels were analyzed on the second flow path using a mobile phase consisting of 50 mM phosphoric acid, 0.5 mM EDTA, 1.6 g/l OSA, 0.5 mM tetramethylammonium chloride, 0.005% Proclin 150 (final concentration), pH 6.5, run at 80 µl/min. After separation on a Acquity UPLC HSS T3 analytical column (1×50 mm, 1.8 µm particle size; Waters, Milford, MA, USA), ACh was enzymatically converted into choline and hydrogen peroxide in a post-column reactor containing immobilized acetylcholine esterase and choline oxidase (Unijet microbore IMER, 1×50 mm; Bioanalytical Systems, Inc., West Lafayette, IN, USA). The hydrogen peroxide generated was then electrochemically detected with a Flex cell (Antec Leyden, The Netherlands) equipped with a glassy carbon electrode (7 mm in diameter) coated with 5 µl of a peroxidase-redox polymer solution (Bioanalytical Systems, Inc.) and operated at +0.1 V versus Pd/H_2_ reference electrode (HyRef™).

The chromatograms were analyzed using the Clarity software package (DataApex, Prague, Czech Republic). The limit of detection was considered as a signal-to-noise ratio (SNR) of 3, corresponding to 50 fmol for ACh and 4 fmol for NA, 5HT.

### Histological analysis

At the end of the microdialysis session, rats were deeply anesthetized with 1.2 ml of pentobarbital (Apoteksbolaget, Stockholm, Sweden) and perfused through the ascending aorta with 50 ml of NaCl 0.9% followed by 250 ml of 4% ice-cold paraformaldehyde (PFA) for 5 mins. The brains were removed and post-fixed for 24 h in 4% PFA and then transferred to 25% sucrose at 4°C for cryoprotection. After 48 h, the brains were sectioned on a freezing microtome at 35 µm thickness in the coronal plane. Sections were collected in 12 series and stored at −20°C in an antifreeze solution made in phosphate buffer containing 30% glycerol and 30% ethylene glycol. Immunohistochemical stainings were performed on free-floating sections using the following antibodies: rabbit anti-TH (1∶5000; P40101-0, Pelfreeze), mouse anti-ChAT (1∶500; MAB305, Millipore), chicken anti-GFP (1∶50000; Ab13970, Abcam), mouse anti-a-syn (1∶100000; syn 211, 36-008, Millipore). The sections were first rinsed in TBS solution (0.5 M Trisma base, 0.15 M NaCl, pH 7.6) followed by quenching of the endogenous peroxidase activity by incubation in 3% H_2_O_2_ and 10% methanol in TBS for 30 min. In order to remove non specific antibody binding, sections were then rinsed in 0.05% Triton-X-100 TBS and incubated for one hour in 0.05% Triton-X-100 TBS containing 5% normal serum matching the species used to raise the corresponding secondary antibodies. Primary antibodies were prepared in 0.05% Triton-X-100 TBS containing 1% bovine serum albumin. For TH and a-syn immunostaining, incubation with primary antibody was performed at room temperature overnight, while sections were incubated with anti-ChAT antibody at 4°C for 48 hours. The sections were then rinsed in 0.05% Triton-X-100 TBS and incubated for one hour at room temperature in 1∶200 dilution of appropriate biotinylated secondary antibody solutions (Vector laboratories), followed by a one-hour incubation in avidin-biotin-peroxidase solution (ABC kit, Vector Laboratories). The staining was visualized using 3,3′-diaminobenzidine (DAB) as a chromogen and 0.01% H_2_O_2_. The sections were then mounted on chromalun-gelatin coated slides, dehydrated in ascending alcohol concentrations, cleared in xylene and coverslipped using DPX (Sigma).

Double immunofluorescent stainings for GFP/ChAT and GFP/TH were performed as described above, with modifications, using primary antibodies at the following concentrations: anti-TH (1∶1000), anti-ChAT (1∶100), anti-GFP (1∶5000). The quenching step was omitted and the peroxidase-based reaction followed by precipitation of DAB replaced by conjugation of a fluorophore, either directly to the secondary antibody or with a streptavidin-biotin amplification step where appropriate (Jackson, Immunoresearch). The sections were directly mounted on glass slides and coverslipped using polyvinyl alcohol-1,4-diazabicyclo[2.2.2]octane (PVA-DABCO®, Sigma). The signal from each fluorophore was captured sequentially using a DMRE laser-scanning microscope equipped with green helium/neon, helium/neon, and argon lasers (Leica, Kista, Sweden).

Insoluble human a-syn positive inclusions were detected by performing a proteinase K (PK) digestion prior to the a-syn immunohistochemical staining [protocol modified from [21]]. Sections were first rinsed in KPBS and incubated at 80°C for 30 min in KPBS. This was followed by a quenching step in 3% H_2_O_2_ and 10% methanol in TBS for 30 min. After further washes, sections were mounted on coated glass slides (Superfrost Ultra Plus, Menzel GmbH) and dried overnight. Sections were then incubated in KPBS containing 10 µg/ml PK (Cat. no 25530-049, InVitrogen) for 5, 30, 60, 90 and 120 min at room temperature. Following washes in KPBS, immunohistochemical staining was performed on slides according to the protocol described above for free-floating sections. Sections obtained from a-syn injected rats were incubated with the human specific mouse anti-a-syn antibody (1∶20000; syn 211, 36-008, Millipore) and control sections obtained from non-injected rats were incubated with a rabbit anti-a-syn antibody that recognizes endogenous rat a-syn (1∶1000; EP1646Y, ab51252, Abcam).

### Stereological analysis

Total ChAT-immunoreactive neurons in the MS/vDBB and total TH-immunoreactive neurons in the VTA and SN were estimated using an unbiased stereological quantification method based on the optical fractionator principle [29]. A low magnification objective (4×) was used to draw the boundaries of the regions of interest, as described previously [15], [23], [30]. Sampling was done using the CAST module in the VIS software (version 4.4.4.0, Visiopharm A/S, Denmark) by an investigator blinded to the identity of the groups. The actual cell counts were obtained with a 60× Plan-Apo oil-immersion objective with a high numerical aperture (NA = 1.4) on a Nikon Eclipse 80i microscope equipped with an *x-y* motorized stage and a *z*-axis motor (Prior, UK). Orientation in the *z*-axis was measured with a Heidenhain microcator. The counting frame was randomly placed on the first counting area and systematically moved through all the counting areas until the entire delineated region was sampled. The *x-y* step length was adjusted so that 150–250 neurons were counted in each delineated region. Estimates of total number of cells were obtained according to the optical fractionator formula. The coefficient of error (CE) attributed to the sampling was calculated according to Gundersen and Jensen [31] and values <0.1 were accepted.

### Statistical analysis

All data are presented as mean ± SEM, unless stated otherwise. All statistical analyses were conducted using the Statistical Package for the Social Sciences version 19 (SPSS Inc., Chicago, IL, USA). To test for left and right side differences in stereological TH counts in SN and VTA and number of pellets taken and eaten in the staircase test, a paired-sample t-test was performed. A one-way ANOVA with a Hochberg's GT2 *post hoc* test used to correct for unequal sample sizes was performed on the stereological (full counts), open field activity test, microdialysis data and mean number of annulus crossings in the probe trial. Two-way repeated measures ANOVA with Greenhouse-Geisser correction for violation of the assumption of sphericity, followed by Tukey HSD *post hoc* test, was performed using the general linear model on the staircase data and the performance over time in the reference memory task. In case of significance and absence of interaction between variables (performance over trial in the working memory task), this was followed by one-way ANOVA coupled to Hochberg's GT2. In cases when Levene's test for unequal variance was significant (average latency in the reference memory test and time spent in the platform quadrant during probe trial), a Welch's Robust ANOVA was performed, followed by a Games-Howell *post hoc* test. In cases where the Shapiro-Wilk test for normality was significant (time savings in the working memory task), a non-parametric Kruskal Wallis test was performed, followed by a Mann Whitney *post hoc* test. Statistical significance was set at 0.05.

## Results

### rAAV5-mediated transgene expression in neurons of the VTA and MS/vDBB

**Figure 2:**
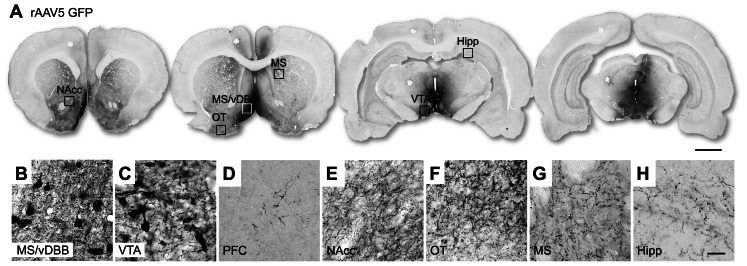
rAAV5-mediated GFP overexpression pattern in the rat brain after injection into the VTA and MS/vDBB. GFP expression was observed in the MS/vDBB as well as throughout the VTA with negligible expression in the SN (A). High-power photomicrographs reveal GFP-immunopositive neurons in the MS/vDBB (B) and the VTA (C). GFP immunoreactivity was visible in fiber projections to the prefrontal cortex (D), nucleus accumbens (E), olfactory tubercule (F), medial striatum (G), and hippocampus (H). Scale bar indicates 2 mm (A) and 30 µm (H). Abbreviations: GFP, green fluorescent protein; Hipp, hippocampus; MS, medial striatum; MS/vDBB, medial septum/vertical limb of the diagonal band of Broca; NAcc, nucleus accumbens; OT, olfactory tubercule; PFC, prefrontal cortex; rAAV5, recombinant adeno associated vectorserotype 5; VTA, ventral tegmental area.

Combined injections of rAAV5 vectors encoding the GFP gene in the VTA and MS/vDBB resulted in expression of the transgene throughout both target nuclei (Fig. 2A). In particular, MS/vDBB and VTA cells were efficiently transduced by the transgene, as demonstrated by a robust labeling of cell bodies in these regions (Fig. 2 B–C). In addition, a widespread GFP immunoreactivity was seen in the fiber terminals of the projection areas, including nucleus accumbens (NAcc), medial striatum, olfactory tubercule, prefrontal cortex and hippocampus, the latter being the main projection area of the MS/vDBB (Fig. 2D–H).

**Figure 3:**
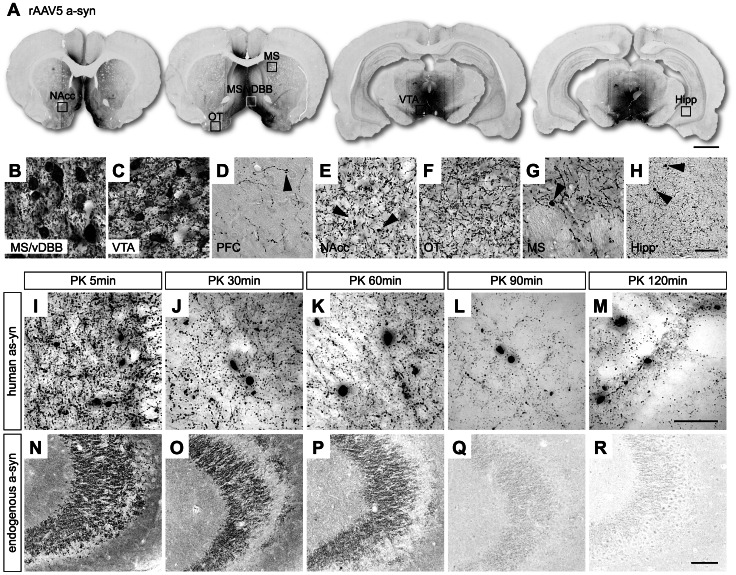
rAAV5-mediated human a-syn overexpression pattern in the rat brain after injection into the VTA and MS/vDBB. Expression of the transgenic a-syn protein was observed in the MS/vDBB as well as throughout the VTA with negligible expression in the SN (A). High-power photomicrographs reveal human a-syn-immunopositive neurons in the MS/vDBB (B) and the VTA (C). Labeled fibers were seen in the prefrontal cortex (D), nucleus accumbens (E), olfactory tubercule (F), medial striatum (G), and hippocampus (H). In addition, human a-syn positive inclusions were seen in the PFC, NAcc, MS and hippocampus (arrow heads in D, E, G, H). Small beaded structures positive for a-syn were also seen along the fibers in the prefrontal cortex (D). PK (10 µm/ml) digestion of hippocampal sections followed by immunostaining specific for human a-syn (clone syn 211) revealed insoluble human a-syn positive aggregates, regardless of incubation time with PK (I–M). Control sections were stained for an antibody that recognizes endogenous rat a-syn (clone EP1646Y) (N–R). Endogenous a-syn expressed in the CA3 region was completely digested after 90 min of incubation with PK (Q–R). Scale bar indicates 2 mm (A), 30 µm (H), 50 µm (M) and 100 µm (R). Abbreviations: a-syn, alpha-synuclein; Hipp, hippocampus; MS, medial striatum; MS/vDBB, medial septum/vertical limb of the diagonal band of Broca; NAcc, nucleus accumbens; OT, olfactory tubercule; PFC, prefrontal cortex; PK, proteinase K; rAAV5, recombinant adeno associated vectorserotype 5; VTA, ventral tegmental area.

In the a-syn group, the extent of expression of the transgene was similar to that of the GFP and covered the MS/vDBB and VTA nuclei (Fig. 3A). Similarly, the human a-syn protein efficiently transduced cells in the MS/vDBB and VTA (Fig. 3B–C). Fiber terminals of the corresponding projection areas showed strong labeling for the transgenic protein (Fig. 3D–H). Contrary to the GFP group, small aggregates were found in the prefrontal cortex, NAcc, medial striatum and hippocampus (Fig. 3D, E, G, H). To determine the solubility of these a-syn inclusions, hippocampal sections were submitted to proteolysis using PK. We found that they were resistant to PK digestion, even after 120 min of incubation, demonstrating the insoluble nature of these aggregates (Fig. 3I–M). In contrast, rat endogenous a-syn expressed at the CA3 level of the hippocampus was completely digested by PK after 90 min of incubation (Fig. 3N–R).

**Figure 4:**
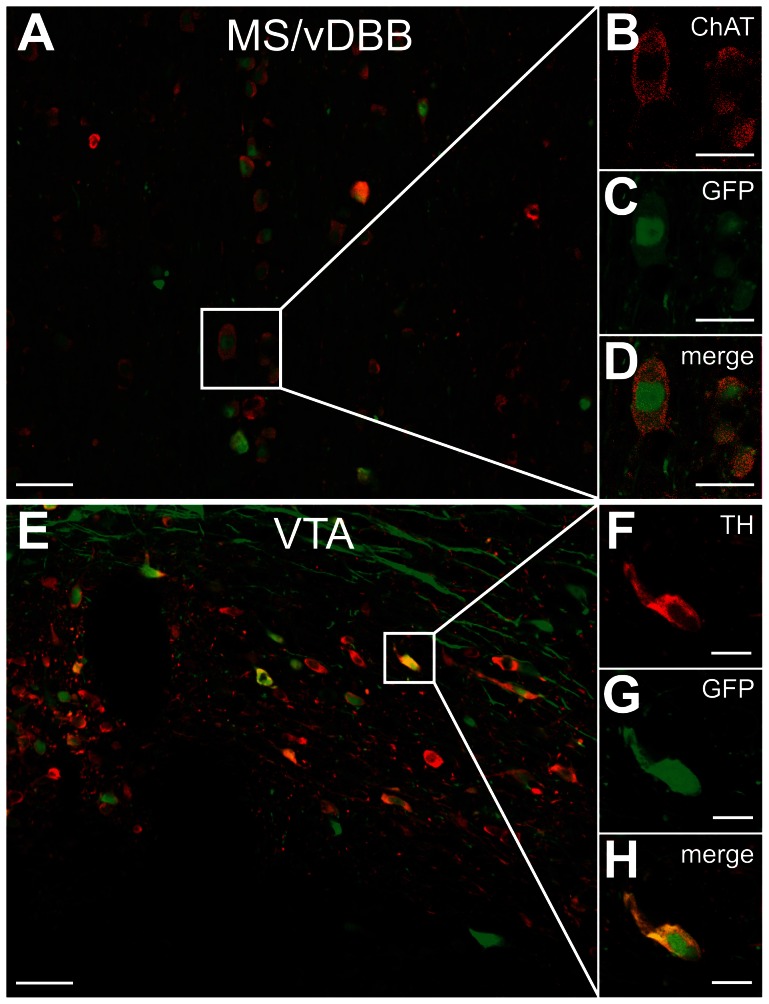
Co-localization of GFP with ChAT or TH after injection of rAAV5 vectors in MS/vDBB or VTA. Confocal microscope images show labeling in the MS/vDBB (A) and the VTA (E), for ChAT (red; B), TH (red; F), GFP (green; C, G) and the merged panels (D, H). Scale bar indicates 50 µm (A, E), 10 µm (B–D) and 5 µm (F–H). Abbreviations: ChAT, choline acetyltransferase; GFP, green fluorescent protein; MS/vDBB, medial septum/vertical limb of the diagonal band of Broca; TH, tyrosine hydroxylase; VTA, ventral tegmental area.

In the present study, we used rAAV5 vectors driving the transgene expression via the synapsin 1 promoter to specifically target neurons. Double immunofluorescence for GFP and specific cholinergic (ChAT) or dopaminergic (TH) phenotypic markers revealed co-localization of the proteins and demonstrated that the rAAV5 vectors were efficient in transducing cholinergic neurons of the MS/vDBB as well as dopaminergic neurons of the VTA (Fig. 4).

### Loss of specific neuronal immunoreactivity induced by rAAV5 vectors

**Figure 5:**
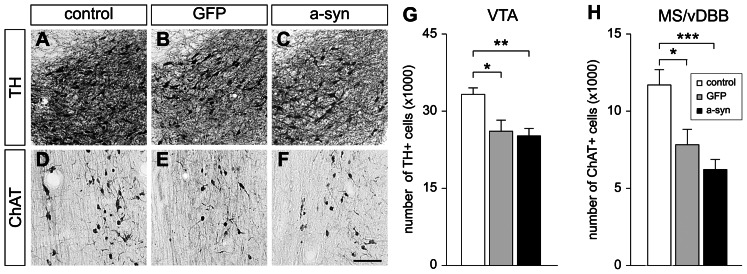
Histological and stereological analyses of cholinergic and dopaminergic neurons. Representative coronal sections from control (A, D), GFP (B, E) and a-syn (C, F) rats, stained for TH in the VTA (A–C) or ChAT in the MS/vDBB (D–F). Overexpression of GFP (B) and a-syn (C) in the VTA induces a reduction in TH-immunoreactivy of 22 and 24%, respectively (G). Overexpression of GFP (E) and a-syn (F) in the MS/vDBB induces a reduction in ChAT-immunoreactivity of 33 and 47% respectively. Scale bar indicates 100 µm. Data are presented as mean ± SEM. * p<0.05; ** p<0.01; *** p<0.001 different from control. (G) One-way ANOVA, F(2, 29) = 7.565, followed by a *post hoc* Hochberg's GT2 test. (H) One-way ANOVA, F(2, 29) = 12.090, followed by a *post hoc* Hochberg's GT2 test. Abbreviations: a-syn, alpha-synuclein; ChAT, choline acetyltrasnferase; GFP, green fluorescent protein; MS/vDBB, medial septum/vertical limb of the diagonal band of Broca; TH, tyrosine hydroxylase; VTA, ventral tegmental area.

To assess the toxic effect of a-syn overexpression in neurons of the VTA and MS/vDBB, the total number of TH- and ChAT-immunoreactive cells in these regions was estimated using computer assisted stereological assessment tools. This analysis showed that the transduction of VTA neurons with a-syn resulted in a mild but significant 24% loss of TH-immunoreactivity compared with control animals (Fig. 5G), as illustrated (Fig. 5A, C). In contrast, the number of TH-positive neurons in the adjacent SN did not change between the a-syn and control groups (23294±1374; 27872±4326, respectively). The deleterious effect of a-syn expression was stronger in the MS/vDBB and induced a 47% loss of ChAT-immunoreactivity compared with control animals (Fig. 5H), as illustrated (Fig. 5D, F). Overexpression of the control GFP protein in the VTA induced a 22% reduction in TH-immunoreactivity (Fig. 5B, G). In addition, transduction of the MS/vDBB neurons with GFP was followed by a 33% reduction in ChAT-immunoreactivity (Fig. 5E, H). These results suggested that the loss of immunoreactivity observed in a-syn expressing animals was not specific to the presence of the human protein. Previous studies have reported non-specific toxicity of high titer rAAV5 vectors used in a range similar to the one used in this study (in the order of 10^13^ genome copies/ml) [24].

### Dopamine-dependent impairment in open field locomotor behavior

**Figure 6:**
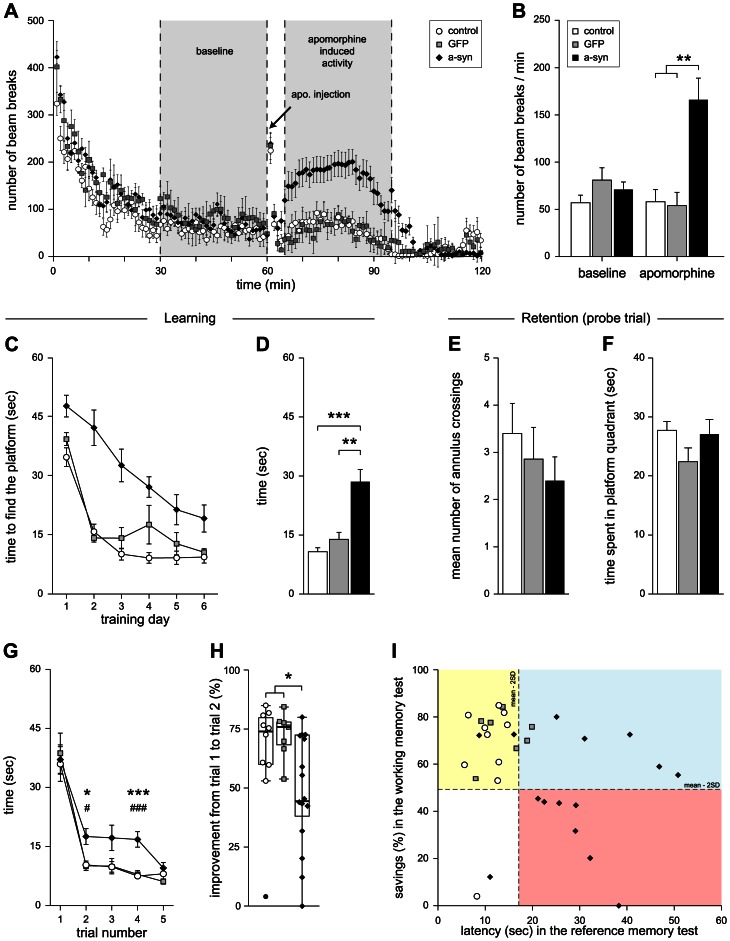
Functional impact of rAAV5-mediated a-syn and GFP overexpression. (A, B) Open field locomotor activity test. Locomotor activity was recorded in the open field test at baseline for 60 min and following apomorphine injection for an additional 60 min (A). Measurements collected between the first 30 to 60 min were averaged to provide an estimation of baseline activity; measurements collected from 5 to 35 min after apomorphine injection were averaged to provide an estimation of the apomorphine-induced activity (B). (C–F) Reference learning and memory performance in the Morris water maze task. Data are presented as average latency over training days (C) and average latency of days 2–6 (D). During the probe trial, mean number of annulus crossings (E) and time spent in the platform quadrant were recorded (F). (G, H) Working memory performance in radial arm water maze task. Data are presented as average latency during days 3–4 over trials (G) and in box plots as percentage of improvement between trials 1 and 2 (time savings, H). (I) Comparison of performance between reference and working memory tasks. Data points represent performance of each individual animal. The vertical dashed line represents the average performance of the control group in the reference memory task (average latency to find the platform) + 2 standard deviations (SD). The horizontal dashed line represents the average performance of the control group in the working memory task (improvement in latency from trial 1 to trial 2 in %) - 2 SD. Data are presented as means ± SEM (B–G), in box plots (horizontal lines represent the 25^th^, 50^th^ -median- and 75^th^ percentiles. Vertical lines extending on either side of the boxes reach the minimum and maximum values within 1.5 inter-quartile ranges. The filled circle indicates an outlier value between 1.5 and 3 interquartile range.) with superimposed individual points (H) or as individual data points (I). * p<0.05; ** p<0.01; *** p<0.001. (B) One-way ANOVA, F(2, 29) = 10.135, followed by a Hochberg's GT2 *post hoc* test. (C) Two-way repeated measures ANOVA, effect of time F(3.773, 109.412) = 48.51, p<0.001; group x time interaction F(7.546, 109.412) = 3.521, p<0.01; group effect F(2, 29) = 15.418, p<0.001; followed by a Tukey HSD *post hoc* test, asyn group different from control (p<0.001) and GFP (p<0.01) groups. (D) Welch's Robust ANOVA, F(2, 15.543) = 14.294, followed by a Games Howell *post hoc* test. (E) One-way ANOVA F(2, 29) = 0.802, p = 0.458. (F) Welch's Robust ANOVA, F(2, 16.2) = 1.825, p = 0.193. (G) Two-way repeated measures ANOVA, effect of trial F(1.982, 57.488) = 61.432, p<0.001; no trial x group interaction F(3.965, 57.488) = 0.998, p = 0.416; group effect F(2, 29) = 5.116, p<0.05; followed by post hoc analysis using one-way ANOVA coupled to Hochberg's GT2. (H) Kruskal Wallis, Chi^2^(2) = 7.428, p = 0.024, followed by a Mann Whitney *post hoc* test.

To determine the effect of a-syn overexpression on the terminals of VTA dopaminergic projections, spontaneous as well as drug-induced locomotion of the animals were assessed in the open-field activity test. No difference in spontaneous locomotion was observed between the groups (Fig. 6A), as shown by similar average numbers of beam breaks/min measured over 30 min under baseline condition in all groups (Fig. 6B). Following administration of a low dose of the DA receptor agonist apomorphine (0.1 mg/kg, sc), the a-syn group exhibited a clear increase in locomotion over a 30 min window compared with both normal and GFP group (Fig. 6A, B).

### Acquisition deficits in reference learning and working memory tasks following overexpression of a-syn

Eight weeks following stereotactic surgery, two aspects of spatial learning and memory were tested using two versions of the water maze task. First, to evaluate the reference memory performance, we subjected the animals to a version of the test in which they needed to use extra maze cues to learn and recall the location of a hidden platform maintained in the same position over consecutive trials and days of training, while their release position in the pool changed at every trial. Control animals (open circles in Fig. 6C) improved their performance over days and quickly learned to locate the platform, as shown by a decrease in latency to find the platform over time. Their performance stabilized after two-to-three days of training, with no further improvement in acquisition detected during the remaining testing days. GFP-treated animals (gray squares) showed similar improvement in their performance across days and were able to rapidly locate the platform. In contrast, overexpression of human a-syn (black diamonds) reduced the ability of the rats to find the escape platform (Fig. 6C). The average latency needed to locate the platform on days 2–6 (corresponding to the plateau performance in control animals) was three times longer in a-syn animals compared with controls (28.6±3.1 and 10.8±1 sec, respectively), whereas the GFP group performed similar to the naive control group (13.9±3.1 sec) (Fig. 6D). At the end of the training session, a probe trial was administered to each group. No difference was found neither in the time spent in the platform quadrant nor in the mean number of annulus crossings (Fig. 6E–F).

Fourty-eight hours after completion of the reference memory test, a version of the task designed to evaluate working memory performance was administered to the animals. A radial arm maze was placed in the water pool and the platform was positioned in alternating arms each of the training days, while the animals were released from different positions at each trial. With this design, the animals have to re-learn the position of the platform every day within five trials by developing a new search strategy. The latency to find the platform was averaged on days 3 and 4 since we found that the animals required 2 days to habituate to the presence of the arms in the pool. On trial 1, all animals showed variable performance, with individual latency to find the platform spreading from 12.5 to 60 sec. On trial 2, control animals reduced the time spent to locate the platform with latencies between 7 and 16.5 sec only. No further improvement was observed on the following (3^rd^ to 5^th^) trials (Fig. 6G). The expression of GFP did not induce any measurable deficit in this test. While the performance of the a-syn overexpressing animals improved between trial 1 and 2, their overall performance over the 5 trials was poorer compared to control animals and the latency to find the platform on trial 2 and 4 was significantly longer compared to both control and GFP animals (Fig. 6G). Time savings, calculated as percentage of improvement from trial 1 to trial 2, were estimated as a measurement of learning efficiency. The median performance of the control and GFP group was 74 and 76%, respectively, while the median performance of the a-syn group was only 45% and significantly lower compared with both control and GFP groups. Note that the control animal that showed a time savings of 4% (filled circle) located the platform in 12.5 sec on trial 1 and 12 sec on trial 2, so that the apparent lack of improvement does not truly reflect that this animal was able to quickly locate the platform on trial 2 (Fig. 6H). The detailed swim paths obtained on the third day of training from representative animals in the three experimental groups are shown in figure S1.

As seen in figure 6I, the performance of all but one control animals in both tasks lie within 2 SD from the mean of the control group (yellow rectangle; upper left corner). Close assessment of the behavior of the control animal whose performance deviated from the mean of the group in the working memory test revealed that this animal managed to immediately locate the platform on the first trial of the test by chance, therefore showing a improvement score from trial 1 to trial 2 close to 0, which does not effectively reflect the overall normal performance of this animal. The data points in the blue rectangle (upper right corner) represent the animals whose performance does not deviate from that one of the controls in the working memory task (within 2 SD from the mean of control animals) but who are impaired in the reference memory task (average latency higher than 2 SD from the mean of the control group). The red rectangle (lower right corner) contains the individual data points of animals impaired in both the working (performance lower than 2 SD from the mean of the control group) and reference memory tasks. Interestingly, the a-syn animals impaired in the working memory test showed co-existing deficits in the reference memory test (red rectangle; lower right corner), while the opposite was not automatically true (blue rectangle; upper right corner) (Fig. 6I).

In order to evaluate whether the learning impairments observed in these animals could be attributed to a deficit in striatum-dependent motor learning, the animals were tested in the paw-reaching task (Fig. S2). With repeated days of testing, all animals improved their performance by increasing the number of pellets taken (two-way repeated measures ANOVA, effect of time, F(2.195, 63.659) = 27.179, p<0.001) and eaten over time (two-way repeated measures ANOVA, effect of time, F(3.457, 100.25) = 63.131, p<0.001), while the number of errors (defined as the number of pellets missed as percentage of the total number of pellets taken) decreased (two-way repeated measures ANOVA, effect of time, F(3.46, 100.351) = 30.575, p<0.001). No significant difference was found in the overall analysis between the groups (two-repeated measures ANOVA, no group x time interaction). These results suggested that all animals were capable of acquiring the motor skills required to perform this striatum-dependent task.

### Extracellular levels of neurotransmitters in the hippocampus

**Figure 7:**
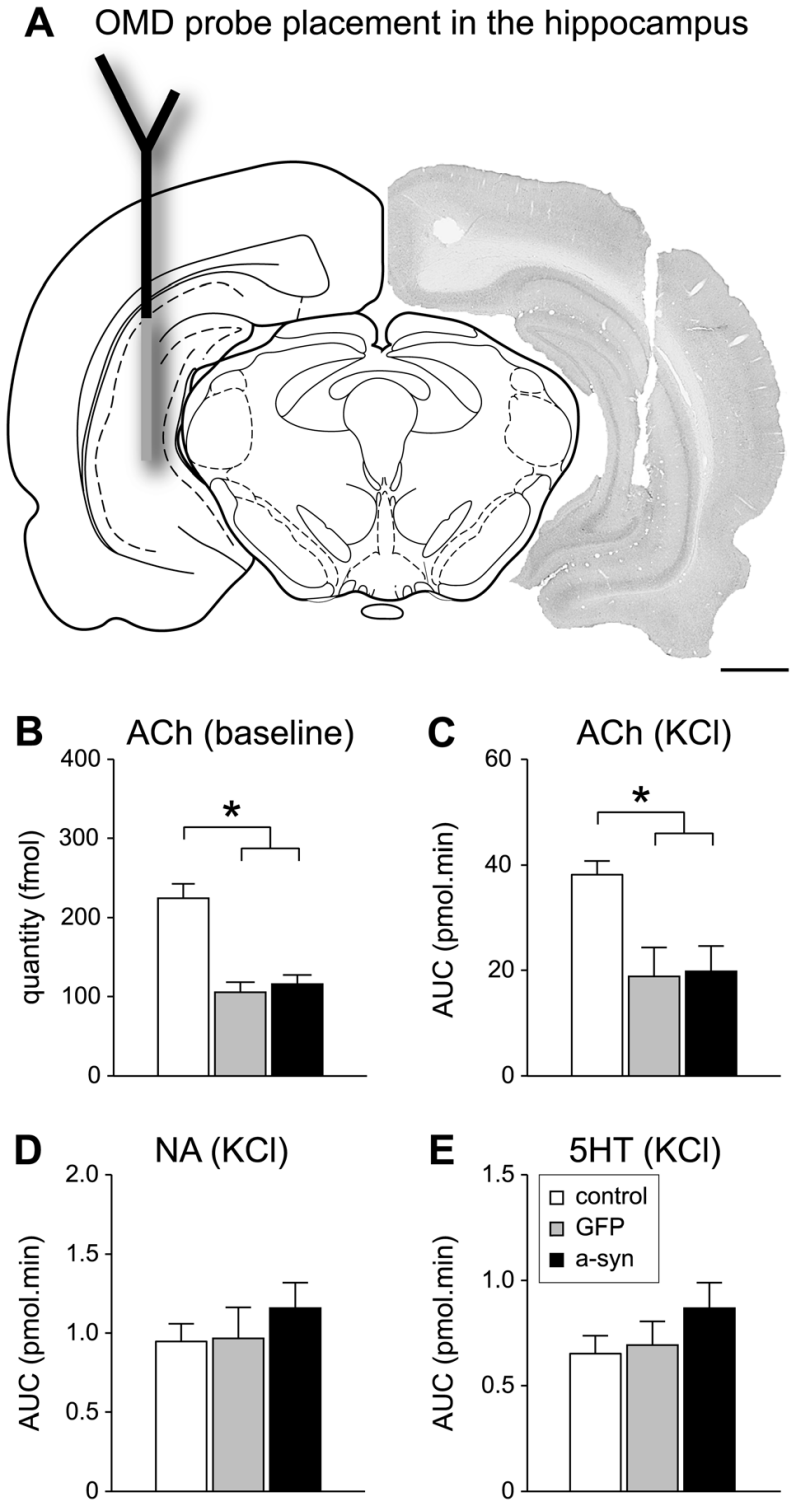
Online microdialysis coupled to HPLC measurements. (A) Representation of a coronal midbrain section showing the target for insertion of the microdialysis probe in the hippocampus (left, adapted from the atlas of Paxinos and Watson [25]) and a probe tract left in the hippocampus after a sampling session (right; section immunostained for ChAT). Both a-syn and GFP groups showed decreased levels of ACh at baseline (B) and a decreased KCl-evoked efflux of ACh (C), compared to controls. KCl-induced release of NA (D) and 5HT (E) was unaffected in GFP and a-syn groups. Scale bar represents 1 mm. Data are presented as mean ± SEM. Baseline levels are average of four baseline samples (in fmol). Amounts of neurotransmitters released after KCl challenge are presented as area under the curve over 24 (NA and 5HT) or 48 (ACh) min after KCl perfusion (in pmol x min). * p<0.05 different from control. (B) One-way ANOVA, F(2, 25) = 5.899, followed by a Hochberg's GT2 *post hoc* test. (C) One-way ANOVA, F(2, 20) = 4.318, followed by a Hochberg's GT2 *post hoc* test. (D) One-way ANOVA, F(2, 23) = 0.614, followed by a Hochberg's GT2 *post hoc* test. (E) One-way ANOVA, F(2, 23) = 1.145, followed by a Hochberg's GT2 *post hoc* test. Abbreviations: ACh, acetylcholine; AUC, area under the curve; NA, noradrenaline; OMD, online microdialysis coupled to HPLC; 5HT, serotonin.

Following completion of the behavioral tests, all three experimental groups were submitted to measurement of extracellular levels of neurotransmitters using an online microdialysis coupled to HPLC detection (OMD) set up for both basal (tonic) and KCl-induced (phasic) release at the level of the hippocampus. The reconfiguration of the OMD system enabled simultaneous analysis of ACh and monoamines (Fig. S3). Four animals were excluded from the final analyses of these data (one animal died during the sampling and three were excluded due to inaccurate probe placement), providing the following numbers of observations in each group: control, n = 10; GFP, n = 5; a-syn, n = 12. Fig. 7A shows the correct placement of the probe in the ventral hippocampus in one of the animals. Probe insertion was followed by a 60-min equilibration phase, after which samples were transferred into each flow path and directly analyzed in twelve-minute intervals. Neostigmine (an ACh esterase inhibitor) was included in the perfusion solutions in order to prevent the rapid extracellular metabolism of ACh. Baseline measurements consisted of an average of four samples. To estimate depolarization-induced neurotransmitter release, KCl was infused for twelve minutes and the corresponding sample together with the previous and next three consecutive samples were used to calculate the total release, estimated by the area under the curve (AUC). The baseline level of ACh in the hippocampus of the intact animals was 225±18 fmol/8 microliters/12 mins and was significantly decreased in a-syn and GFP expressing animals (by 48 and 53%, respectively) (Fig. 7B). Addition of KCl to the perfusion fluid resulted in increased extracellular levels of ACh in intact animals (38±2.6 pmol.min). The ACh release stimulated by KCl was significantly reduced to 20±4.8 pmol.min in the a-syn group (48% decrease) and 19±5.5 pmol.min in the GFP group (51% decrease) (Fig. 7C). These changes in ACh release appeared to be specific as the KCl-induced release of both NA and 5HT measured at the same time were not affected in either of the transgene overexpressing groups, compared with naive controls (Fig. 7D, E).

## Discussion

The aim of this study was to assess the effects of combined targeted overexpression of human wild-type a-syn in the VTA and MS/vDBB in rats using rAAV5 vectors. Both dopaminergic neurons in the VTA and cholinergic neurons in the MS/vDBB were efficiently transduced by this serotype of AAV vector. Importantly, overexpression of the a-syn protein led to the emergence of dystrophic fibers and formation of aggregates in projection areas (particularly in the NAcc and hippocampus). PK treatment showed that these aggregates were insoluble, as observed in PD patients [32]. This was associated with the development of DA-dependent motor disturbances as well as spatial learning and memory deficits in reference learning and working memory tasks.

Our results suggested that overexpression of a-syn did not result in specific neurodegeneration of either the dopaminergic neurons in the VTA or the cholinergic neurons in the MS/vDBB, in contrast with its effects on nigral dopaminergic neurons [18], [19], [20]. A previous study using rAAV2-A53T a-syn vectors injected in the VTA showed that the mesocorticolimbic projections were pathologically affected, with presence of aggregates and dystrophic neurites [23]. However, the dopaminergic VTA neurons were resistant to rAAV2-mediated overexpression of a-syn, while the same vector did cause prominent cell loss in the dopaminergic nigral neurons when injected in the neighboring SN. In addition, the animals expressing a-syn showed an abnormal locomotor activity in DA-dependent behaviors, thus highlighting that neurons surviving following overexpression of a-syn could nonetheless become dysfunctional and contribute to the development of behavioral deficits [23]. In our study, the partial loss of ChAT- and TH-immunoreactivity reported after rAAV5 vector injection is likely due to non-specific effects of the vector as demonstrated by the loss of immunoreactivity in the GFP group similar to the one observed in the a-syn group. In fact, toxicity of the GFP protein has been reported in other studies and is known to be dose-dependent [24], [33]. Furthermore, it is likely that this effect is serotype, species and cell type specific. Nonetheless, a-syn overexpression-dependent behavioral effects were observed. In particular, the a-syn animals responded selectively to the injection of apomorphine, a DA agonist, by an increase in general locomotor activity, demonstrating a supersensitivity of post-synaptic receptors. Interestingly, whereas direct gene delivery using viral vector to overexpress a-syn in nigral neurons is associated with severe neurodegeneration and motor dysfunction in rats [18], [34], a-syn transgenic mice displaying aggregation of a-syn in neuronal bodies and processes can develop motor deficits in the absence of neuronal loss [35], [36], suggesting that neuronal dysfunction, more so than neuronal degeneration, may be sufficient to trigger the onset of certain behavioral deficits. Our results are consistent with this hypothesis and imply that the response to apomorphine seen in the a-syn group is most likely driven by a terminal dysfunction at the ventral striatal level, as suggested by the presence of inclusions at that level.

The majority of the available animal models of PD only mimics the motor symptoms of the disease and do not replicate the heterogeneous aspect of the symptoms. However, recent studies have described a-syn transgenic mice presenting cognitive disturbances [37], [38], [39], therefore echoing studies in humans showing an association between LB and cognitive impairment in PD [40]. In particular, cognitive deficits have been detected in the Y-maze, novel object recognition and operant reversal learning tests in transgenic mice overexpressing human wild-type a-syn under the Thy1 promoter [38]. These mice exhibit human a-syn expression in cholinergic neurons of the basal forebrain, a reduction in ACh level in the cerebral cortex [38], as well as alteration in striatal DA release and reduction in striatal TH expression [41]. In addition, tetracycline-controllable a-syn transgenic mice under the CaMKIIα promoter have shown deficits in learning and memory in the Morris water maze task [42]. Importantly, abnormal accumulation of a-syn was detected in limbic regions (including the hippocampus) of these mice [37].

Although a-syn transgenic mice models clearly demonstrate a connection between increased levels of a-syn and development of cognitive deficits, these models are accompanied by widespread accumulation of a-syn. This makes it difficult to dissect the neurobiological basis underlying the development of each specific symptom. There is currently no documented report of the effects of targeted a-syn overexpression in non-nigral dopaminergic neurons as well as non-dopaminergic neurons on learning and memory, despite their possible involvement in mediating cognitive deficits in PD. In a study based on toxin-induced lesion in rats, injection of 6-OHDA in the VTA resulting in a loss of 50% of DAergic neurons in that region was sufficient to elicit impairment in a reference memory task. Simultaneous lesion of 90% of the cholinergic neurons in the MS/vDBB following 192IgG-saporin injection in that region resulted in additional deficit in a working memory task. Taken together, these results suggested that the integrity of both mesocorticolimbic DAergic and septohippocampal cholinergic pathways might be required to regulate certain aspects of memory in rats [15]. Following targeted overexpression of human wild-type a-syn in these regions in rats, our findings suggested that similar behavioral deficits could be obtained independently of dopaminergic and cholinergic cell loss and that severe a-syn pathology in target regions such as the hippocampus may induce substantial effect on behavior. Two recent studies have looked at the septohippocampal cholinergic pathway in mutant A30P a-syn transgenic mice under the Thy1 promoter and at the effects of additional DA deficiency. Interestingly, the mice displayed a delayed loss of cholinergic neurons in the MS/vDBB in response to DA depletion following chronic MPTP administration, suggestive of a link between a-syn load, DA depletion and cholinergic dysfunction [43], [44].

Deficits in learning and memory observed in a-syn transgenic mice are accompanied by alterations in post-synaptic densities [39] or reduction in presynaptic vesicle proteins such as synaptophysin [37], suggesting that the toxicity of a-syn may be mediated by synaptic defects, a hypothesis endorsed by studies in cell culture [45], [46]. Impaired synaptic neurotransmission, characterized by profound reduction in presynaptic striatal DA release and reuptake, has also been documented *in vivo* in rats overexpressing human wild-type a-syn in nigral dopaminergic neurons, using amperometry [47]. Interestingly, since we measured marked reduction in the basal and KCl-evoked release of extracellular ACh in the hippocampus of both a-syn and GFP animals, the unique behavioral impairment observed in the a-syn animals did not appear to correlate with the deficit in neurotransmitter release measured by OMD. However, we cannot infer that the dopaminergic neurotransmission in the hippocampus was not affected since we were unable to measure DA levels at a satisfactory SNR level. It should be pointed out that no other structures, which may be relevant to the behavioral impairment observed in the animals, were sampled by OMD in this study. Moreover, one of the recognized caveats of OMD is the low time resolution of this technique, typically in the order of 10 minutes, which contrasts to amperometry, a method that can monitor release and reuptake dynamics of neurotransmitter in small areas in the order of seconds.

Our study showed that targeted overexpression of human a-syn restricted to the VTA and MS/vDBB in rats was sufficient to induce deficits in learning and memory that may reminisce to some extent some of the early cognitive deficits seen in humans. This was associated with the presence of abundant insoluble a-syn positive aggregates in the mesocorticolimbic and septohippocampal pathways. Although the toxicity of high-titer AAV5 vectors will need to be addressed further by performing dose-dependent experiments, this study constitutes nonetheless a first attempt at modeling spatial learning and memory deficits in rats by overexpression of wild type human a-syn in the VTA and MS/vDBB and may contribute to further understanding how cognitive deficits emerge in PD patients.

## Supporting Information

Figure S1 **Search strategies adopted during the working memory test.** The swim paths taken by representative animals from the different groups at day 3 of training are illustrated. The grey circles show the position of the hidden platform and the red dots point at the release position of the animals into the pool, from alternating arms (numbered 1 to 6) over five trials. (A) On trial 1, the control rat was actively searching for the escape platform in three arms of the maze, without reattempting to explore already visited arms. The animal also spent time in the middle portion of the maze but did not locate the platform within the 60 sec of the trial (therefore it was gently guided towards it and allowed to remain on it 20 sec). Note that the platform was positioned in arm 2 on day 1 and in arm 1 on day 2. On trial 2, the animal was capable of orienting towards the platform very rapidly and swam straight to the platform after being released into the pool. The swim behavior on the following three trials remained identical. (B) The behavior of the GFP rat on trial 1 resembled that of the control animal. Unlike the control rat though, the GFP rat found the platform while exploring arm 5. On the following trial, it swam straight to the platform and maintained a low latency to find the platform on the remaining trials. It first swam into a different arm on trial 3 and 4 but was able to quickly readjust its direction to swim to the platform, as shown by the sharp turns made at the end of arms 3 and 4. (C) The a-syn treated rat, on the other hand, performed poorly across all trials and did not show any improvement. Although it explored arm 5 and found the platform on the first trial, it explored all arms but arm 5 on trial 2 to 4 and persisted in visiting already explored arms that did not contain the platform, demonstrating that this animal was not capable of quickly adapting a successful search strategy as the other animals did.
(TIF)

Figure S2 **Assessment of striatum-dependent motor learning using the staircase test.** At the beginning of each daily session throughout the eleven-day test period, both sides of the staircase were baited with 40 sugar pellets, equally distributed between four steps. At the end of the 20-min session, the total number of pellets taken (A) and eaten (B) were counted and the errors calculated as the total number of failed attempts as percentage of the total number of pellets taken (C).
(TIF)

Figure S3 **Typical chromatograms of monoamines and acetylcholine standards analyzed with reconfigured online microdialysis coupled to HPLC setup and methods.** A mixture of all standards were injected at the individual concentrations of 10 nM for 3,4-dihydroxy-L-phenylalanine (L-DOPA), noradrenaline (NA), 3,4-dihydroxyphenylacetic acid (DOPAC), dopamine (DA), homovanillic acid (HVA), serotonin (5-HT) and 3-methoxytyramine (3-MT), 100 nM for indoleacetic acid (5-HIAA) and 300 nM for ACh. The specificity of the methods and instrument configuration enables parallel analysis of all individual compounds with one single injection split into two loops. (A) The monoamines were analyzed using a reversed phase chromatographic separation coupled with electrochemical detection, allowing analysis of eight monoamines at complete baseline separation within ten minutes. (B) Acetylcholine (ACh) was analyzed using reversed phase chromatographic separation followed by a dual step enzymatic reactor coupled to enzymatic sensor electrode detection. (See method section for detailed description).
(TIF)
