# Supplementary figures and images for: Characterization of Cognitive Deficits in Rats Overexpressing Human Alpha-Synuclein in the Ventral Tegmental Area and Medial Septum Using Recombinant Adeno-Associated Viral Vectors
Source: PLoS One. 2013 May 21;8(5):e64844. doi: 10.1371/journal.pone.0064844 (PMC3660601; doi:10.1371/journal.pone.0064844)

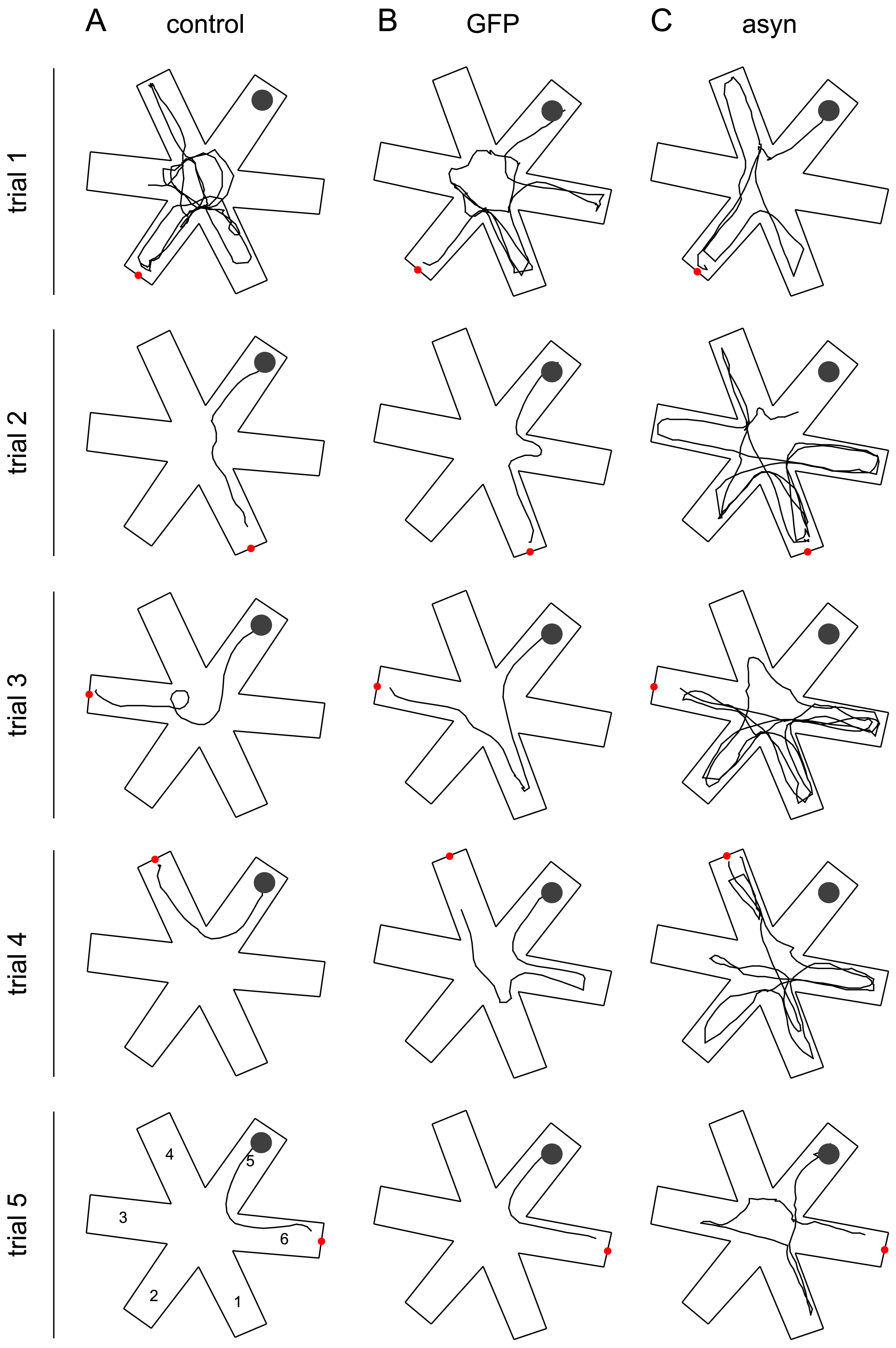

Supplement: Figure S1 — Search strategies adopted during the working memory test. The swim paths taken by representative animals from the different groups at day 3 of training are illustrated. The grey circles show the position of the hidden platform and the red dots point at the release position of the animals into the pool, from alternating arms (numbered 1 to 6) over five trials. (A) On trial 1, the control rat was actively searching for the escape platform in three arms of the maze, without reattempting to explore already visited arms. The animal also spent time in the middle portion of the maze but did not locate the platform within the 60 sec of the trial (therefore it was gently guided towards it and allowed to remain on it 20 sec). Note that the platform was positioned in arm 2 on day 1 and in arm 1 on day 2. On trial 2, the animal was capable of orienting towards the platform very rapidly and swam straight to the platform after being released into the pool. The swim behavior on the following three trials remained identical. (B) The behavior of the GFP rat on trial 1 resembled that of the control animal. Unlike the control rat though, the GFP rat found the platform while exploring arm 5. On the following trial, it swam straight to the platform and maintained a low latency to find the platform on the remaining trials. It first swam into a different arm on trial 3 and 4 but was able to quickly readjust its direction to swim to the platform, as shown by the sharp turns made at the end of arms 3 and 4. (C) The a-syn treated rat, on the other hand, performed poorly across all trials and did not show any improvement. Although it explored arm 5 and found the platform on the first trial, it explored all arms but arm 5 on trial 2 to 4 and persisted in visiting already explored arms that did not contain the platform, demonstrating that this animal was not capable of quickly adapting a successful search strategy as the other animals did. (TIF) [file pone.0064844.s001.tif]

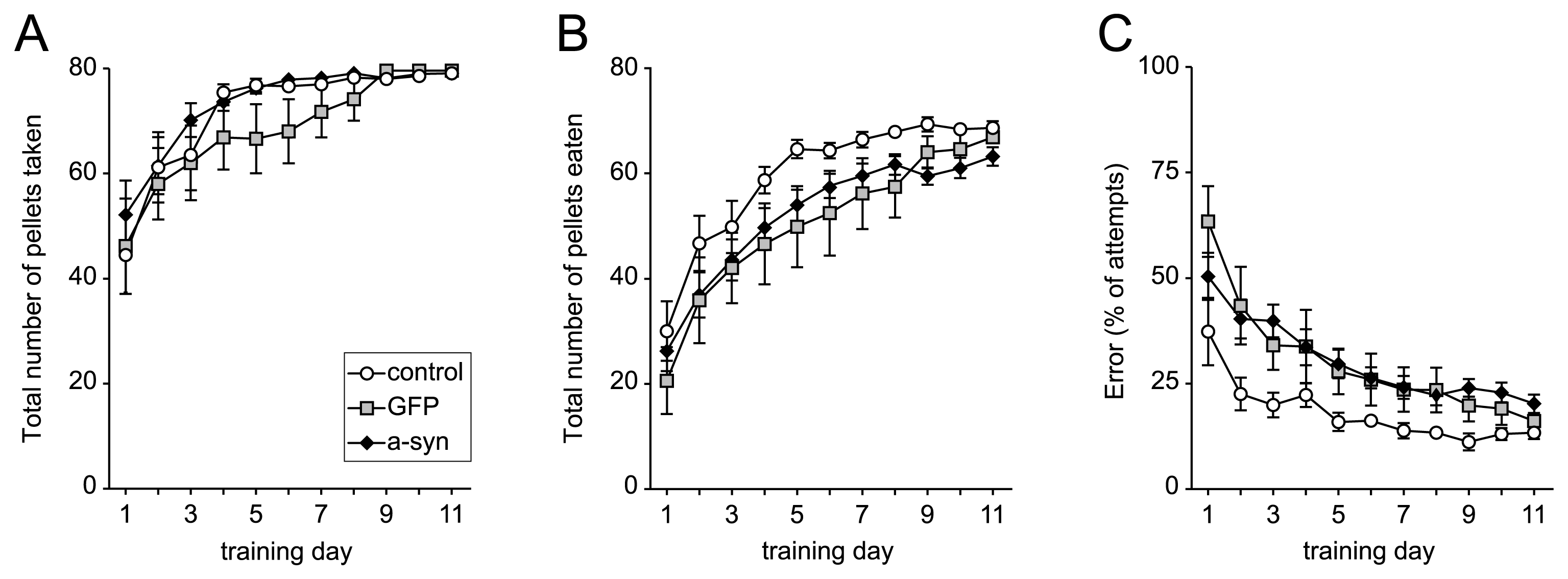

Supplement: Figure S2 — Assessment of striatum-dependent motor learning using the staircase test. At the beginning of each daily session throughout the eleven-day test period, both sides of the staircase were baited with 40 sugar pellets, equally distributed between four steps. At the end of the 20-min session, the total number of pellets taken (A) and eaten (B) were counted and the errors calculated as the total number of failed attempts as percentage of the total number of pellets taken (C). (TIF) [file pone.0064844.s002.tif]

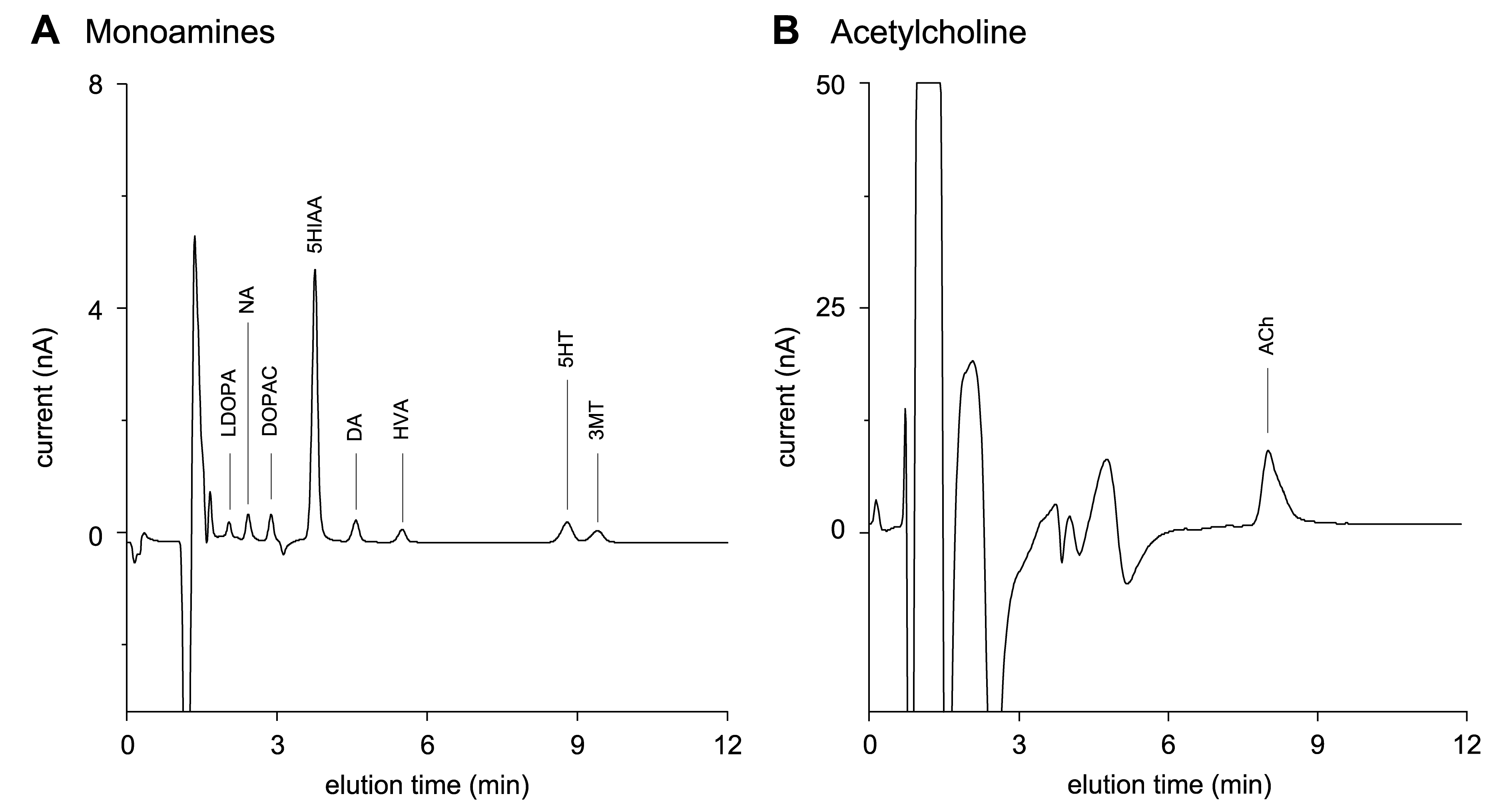

Supplement: Figure S3 — Typical chromatograms of monoamines and acetylcholine standards analyzed with reconfigured online microdialysis coupled to HPLC setup and methods. A mixture of all standards were injected at the individual concentrations of 10 nM for 3,4-dihydroxy-L-phenylalanine (L-DOPA), noradrenaline (NA), 3,4-dihydroxyphenylacetic acid (DOPAC), dopamine (DA), homovanillic acid (HVA), serotonin (5-HT) and 3-methoxytyramine (3-MT), 100 nM for indoleacetic acid (5-HIAA) and 300 nM for ACh. The specificity of the methods and instrument configuration enables parallel analysis of all individual compounds with one single injection split into two loops. (A) The monoamines were analyzed using a reversed phase chromatographic separation coupled with electrochemical detection, allowing analysis of eight monoamines at complete baseline separation within ten minutes. (B) Acetylcholine (ACh) was analyzed using reversed phase chromatographic separation followed by a dual step enzymatic reactor coupled to enzymatic sensor electrode detection. (See method section for detailed description). (TIF) [file pone.0064844.s003.tif]
